# Land Use Regression Modeling of Outdoor Noise Exposure in Informal Settlements in Western Cape, South Africa

**DOI:** 10.3390/ijerph14101262

**Published:** 2017-10-20

**Authors:** Chloé Sieber, Martina S. Ragettli, Mark Brink, Olaniyan Toyib, Roslyn Baatjies, Apolline Saucy, Nicole Probst-Hensch, Mohamed Aqiel Dalvie, Martin Röösli

**Affiliations:** 1 Department of Epidemiology and Public Health, Swiss Tropical and Public Health Institute, Basel 4002, Switzerland; chloe.sieber@gmail.com (C.S.); martina.ragettli@unibas.ch (M.S.R.); apolline.saucy@gmail.com (A.S.); nicole.probst@unibas.ch (N.P.-H.); 2 University of Basel, Basel 4003, Switzerland; 3 Federal Office for the Environment, CH-3003 Bern, Switzerland; mark.brink@bafu.admin.ch; 4 Centre for Environmental and Occupational Health Research, School of Public Health and Family Medicine, University of Cape Town, Rondebosch, Cape Town 7700, South Africa; olaniyanolan@gmail.com (O.T.); aqiel.dalvie@uct.ac.za (M.A.D.); 5 Department of Environmental and Occupational Studies, Faculty of Applied Sciences, Cape Peninsula University of Technology, Cape Town 7700, South Africa; baatjiesr@cput.ac.za

**Keywords:** noise measurement, road traffic noise, neighborhood noise, land use regression, informal settlements, low- and middle- income country, South Africa

## Abstract

In low- and middle-income countries, noise exposure and its negative health effects have been little explored. The present study aimed to assess the noise exposure situation in adults living in informal settings in the Western Cape Province, South Africa. We conducted continuous one-week outdoor noise measurements at 134 homes in four different areas. These data were used to develop a land use regression (LUR) model to predict A-weighted day-evening-night equivalent sound levels (L_den_) from geographic information system (GIS) variables. Mean noise exposure during day (6:00–18:00) was 60.0 A-weighted decibels (dB(A)) (interquartile range 56.9–62.9 dB(A)), during night (22:00–6:00) 52.9 dB(A) (49.3–55.8 dB(A)) and average L_den_ was 63.0 dB(A) (60.1–66.5 dB(A)). Main predictors of the LUR model were related to road traffic and household density. Model performance was low (adjusted R^2^ = 0.130) suggesting that other influences than those represented in the geographic predictors are relevant for noise exposure. This is one of the few studies on the noise exposure situation in low- and middle-income countries. It demonstrates that noise exposure levels are high in these settings.

## 1. Introduction

Noise exposure can lead to auditory and non-auditory health effects [1]. Non-auditory health effects include, namely, annoyance [2], sleep disturbance [3], cardiovascular diseases [4,5,6,7], diabetes [8,9], depression [10,11], and impairment of cognitive performance [12,13,14,15]. In 2011, the World Health Organization (WHO) reported that about 50% of the European urban population was exposed to road traffic noise levels (day-evening-night equivalent sound level, L_den_) above 55 A-weighted decibels (dB(A)), leading to 490,000 Disability-Adjusted Life Years (DALYs) lost every year due to road traffic annoyance. When including railway noise and aircraft noise, annoyance related DALYs increase up to about 654,000 DALYs. Additionally, 22,000 DALYs, 45,000 DALYs, 61,000 DALYs, and 903,000 DALYs are due to tinnitus, cognitive impairment of children, ischemic heart disease, and sleep disturbance, respectively [16]. Therefore, research on noise exposure is crucial, especially since urbanization is expanding in many countries around the world [17]. 

In North America, in Europe, and in some Asian countries numbers of studies on noise exposure and/or its related health effects have been conducted [1]. In low- and middle-income countries few studies addressing this issue have been carried out. Nonetheless, a prerequisite to explore the association between noise exposure and noise effects on health is a proper exposure assessment of noise levels. In Chile, a low-cost, vehicular traffic noise predictive model has been used to evaluate noise levels in the city of Santiago de Chile [18]. Apart from a Nigerian study [19] that compared noise levels in different settings, little information from African countries can be found in the literature. A challenge in these countries is the availability of suitable noise emission data, which would be needed for propagation modeling. In such conditions, land use regression (LUR) modeling may be used as a substitute to empirically assess the relation between noise levels and topographical predictors at given locations. Once established, such a LUR model may be used to predict noise levels at other positions, where no noise measurements were realized, but where geographic data are available. This method has mainly been used to develop air pollution models, but it has proved its ability to model spatial patterns of noise levels within large areas and cities in different regions: e.g. the Dalian Municipality, Girona, Grenoble, Basel, or Montreal [17,20,21]. Being able to model noise in low- and middle-income countries would be a palliative solution to the difficulty of obtaining a sufficient number of noise measurements to assess general outdoor noise exposure.

The present study aimed to investigate the overall noise exposure of sites spread in four different informal settings of the Western Cape, South Africa. The objective was to develop a LUR model using one-week outdoor noise measurements and geographical land use data to assess the spatial variability of environmental noise levels. 

## 2. Materials and Methods 

### 2.1. Study Design and Study Areas

As part of a health study designed as a longitudinal cohort study on air pollution and respiratory health outcomes among children in informal settings in the Western Cape province, South Africa, outdoor noise levels were measured in parallel with air pollution [22,23]. These measurements were carried out at a representative number of children homes located in four areas including Khayelitsha, Marconi-Beam, Oudtshoorn, and Masiphumulele during the South African summer in 2015–2016. Khayelitsha is an impoverished peri-urban area with a large informal sector that has about 391,749 inhabitants, 10,120 persons/km^2^, and an average of 3.2 persons/household [24]. Marconi-Beam is an informal settlement located in an urban industrialized area that houses a petrochemical refinery, and has about 95,630 inhabitants, 2189 persons/km^2^, and an average of 2.7 persons/household [25]. Oudtshoorn is a rural informal settlement that has about 29,143 inhabitants, 870 persons/km^2^, and an average of 3.4 persons/household [26]. Masiphumulele is the area with the least exposure to road traffic, as well as to industrial emissions, and counts about 4424 inhabitants, 1101 persons/km^2^, and an average of 2.5 persons/household [27]. Households, where noise measurements were collected, were selected for the respiratory health study, based on their location, and their expected air pollution exposure in order to have a sample covering the whole air pollution range over each area.

### 2.2. Data Collection and Data Treatment

#### 2.2.1. Noise Exposure Measurements

The initial aim was to conduct one-week outdoor noise measurements at 40 homes of school children in Khayelitsha, Marconi-Beam, and Oudtshoorn, and at 20 homes in Masiphumulele. We scheduled four consecutive one-week outdoor noise measurements at two schools in Khayelitsha, and in Oudtshoorn, as well as at one school in Marconi-Beam, and in Masiphumulele (Supplementary Materials, Figure S1). In addition, we planned four consecutive one-week outdoors noise measurements at one reference site in each area, where the South African government itself conducted air pollution monitoring. Each sampling site was geocoded using a Global Positioning System (GPS). All the measurements took place between 9 November 2015 and 10 May 2016. The setups (≤10 per day) were performed on either a Monday or a Tuesday, and the removals (≤10 per day), approximately seven days later on either a Monday or a Tuesday. 

We used a Noise Sentry RT type-II sound level meter data logger (Convergence Instruments, Sherbrooke, QC, Canada) installed outside each location to measure A-weighted equivalent sound pressure levels (L_Aeq_) averaged at one-second intervals continuously over seven days. The noise meters were calibrated before each measurement. We mounted them on a pole that we usually attached to a fence or on part of the home, which was not directly affected by a local source (e.g., air conditioning). The noise meters were fixed at least one meter away from the roof and the wall to avoid noise reflection. 

For the analysis, we restricted noise measurements to five successive days, from Wednesday at 06:00 to Monday at 06:00, to have the same measurement days for each site. Samples with data missing for more than 10% of the time (due to technical issues such as battery failure) were excluded from the analyses. We also removed outliers, defined as one-second noise measurements exceeding the five-day mean by plus or minus three standard deviations.

Using the cleaned data, we computed various A-weighted equivalent sound level variables: L_day_ (06:00–18:00), L_evening_ (18:00–22:00), L_night_ (22:00–06:00), L_Aeq24h_ (06:00–06:00 on the next day), and L_den_ which is comparable to L_Aeq24h_, but with 5 dB penalty for the evening measurements and 10 dB penalty for the night measurements. We favored the noise metrics starting at 6:00 and not 7:00 in the morning because in South Africa daily activities begin and end earlier than in many European countries.

#### 2.2.2. Noise Exposure Predictor Variables

For the development of the LUR model, we collected geographic information data potentially contributing to noise levels. The City of Cape Town and the Municipality of Oudtshoorn provided us with roads and railway networks, airport and community service positions, household density, as well as land use, all obtained through geographic information systems (GIS). Detailed source information is provided in Table S1. Based on the type of the roads, and on the presumed traffic according to our personal knowledge of the areas, we classified them into four categories: large roads for national roads (highways); medium roads for metropolitan, provincial, and regional roads; small roads for local roads; and very small roads for neighborhood roads. From these data and using the program ArcGIS (ArcGIS 10.3.2, ESRI, Redlands, CA, USA) we computed for each sampling site several variables potentially influencing noise levels (Table 1). The normalized difference vegetation index (NDVI), a substitute for green spaces, was also computed using ArcGIS, based on Landsat 8 images acquired from the U.S. Geological Survey website [28]. The picture selected for Khayelitsha, Marconi-Beam, and Masiphumulele dated from the 1 January 2016 and had a cloud coverage <10%, and the one for Oudtshoorn dated from the 31 March 2016 and had a cloud coverage <20%. 

### 2.3. Statistical Analyses

#### Development of the LUR Model for Noise Prediction

We developed a LUR model for the overall noise exposure of the four areas together to explain L_den_ at 134 measurement sites. The noise metric L_den_ was used because it is considered to represent best the noise burden and, thus, the noise sensitivity and noise annoyance allowing us further application of the model [29]. We first carried out univariate analyses with all the GIS predictor variables listed in Table 1. For each variable type, we selected its best buffer size, based on the sign of its coefficient, and the R^2^ of the model. We then performed a stepwise forward selection with these variables. We added them one by one in the model, starting with the variable which obtained the highest R^2^ in the univariate analysis [21]. Only variables having a correlation value of <0.7 with the variables already in the model, and leading to an increase of the adjusted R^2^ were kept. Once adding additional variables did not improve the model anymore, the variables with a *p*-value > 0.2 were removed one by one. We then challenged each variable already in the model with all the variables and all their different buffer sizes used in the univariate analyses to test if any of them could better explain the measured noise levels. Subsequently, we tested if the addition of one of the aforesaid variables improved the LUR model. The supplementary variable was kept in the model only if its *p*-value was ≤0.2. The model with the best adjusted R² was retained. 

## 3. Results

In total, 134 valid long-term outdoor noise measurements were obtained from 127 households, five schools, and one reference site, which corresponds to the official air pollution measurement site of the community (Figure S1). In Khayelitsha, 42 outdoor noise measurements were collected (36 at the homes, two at two different schools selected, one at the reference site), 37 were conducted in Marconi-Beam (35 at the homes, one at the selected school, one at the reference site), 39 in Oudtshoorn (38 at the homes, one at the selected school), and 16 in Masiphumulele (15 at the homes, one at the selected school). Four to five weeks of noise measurements were conducted at the schools, and at the reference sites, but one measurement week per site being enough for the analyses only the first one was kept.

Table 2 displays the summary statistics of each of the noise measurements periods (L_day_, L_evening_, L_night_, L_Aeq24h_, and L_den_) computed from the cleaned noise measurement data across all sites. Figure S2 in the Supplementary Materials illustrates these noise metrics by means of boxplots for each study area. Mean L_den_ were 63.8 dB(A), 64.2 dB(A), 60.8 dB(A), 63.4 dB(A) in Khayelitsha, Marconi-Beam, Oudtshoorn, and Masiphumulele, respectively.

A LUR model was developed for L_den_ (Table 3). The potential predictor variables, their corresponding best buffer, and the results of the univariate analyses with L_den_ are provided in the Table S2. The final LUR model contains a total of five relevant variables—two variables related to road traffic, one to the household density, and two to the land use (commercial and industrial). The relationship between L_den_ and the independent variables was, however, weak (adjusted R^2^ = 0.130). This was also reflected in the large value of the intercept (61.30 dB(A)), and the low coefficient value of the independent variables. The summary statistics of the model variables is provided in Table S3 in the Supplementary Materials.

**Figure 1:**
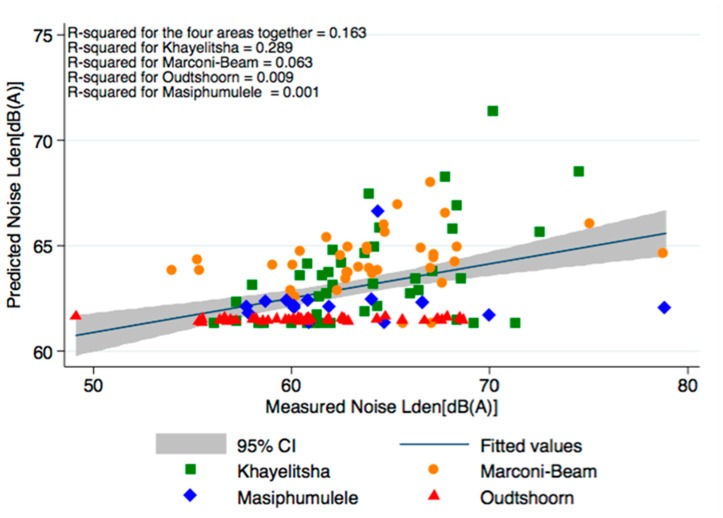
Scatter plot of the predicted noise (L_den_) against the measured noise (L_den_), with different symbols for each of the four study areas (Khayelitsha with n = 42, Marconi-Beam with n = 37, Masiphumulele with n = 16, and Oudtshoorn with n = 39). The fitted value line and the 95% confidence interval (grey zone) are also represented, as well as the R^2^ giving the relationship between the noise predicted and the noise measured.

**Figure 2:**
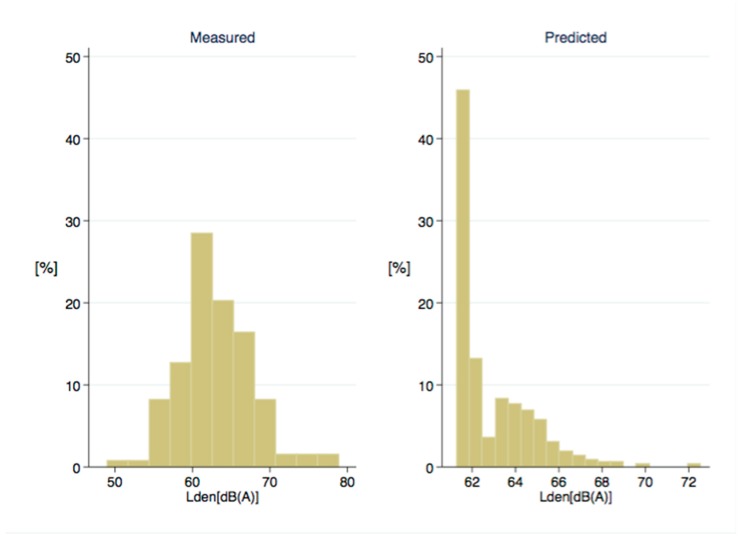
Distribution in percentages (%) of the measured (**left**) noise (n = 134) and predicted (**right**) levels (L_den_) (n = 364).

Figure 1 depicts a weak relationship (R^2^ = 0.163) between the noise measured and the noise predicted at the measurement sites using our LUR model. Nevertheless, differences can be seen between the four study areas. Highest correlation was found in Khayelitsha (R^2^ = 0.289), followed by Marconi-Beam (R^2^ = 0.063), Oudtshoorn (R^2^ = 0.009), and Masiphumulele (R^2^ = 0.001). Figure 2 describes the distribution of the measured and predicted noise levels at the 134 sites and 364 sites, respectively. Figure S3 shows the distribution of measured and predicted noise levels (L_den_) for each study area separately using boxplots. In each area, medians of measured and predicted noise levels are similar, but lower data variability for the latter one is observed. Three graphs (Figures S4–S6) in Supplementary Materials depict the residuals of the noise model. There is no relationship between the residuals and the fitted values (Figure S4), and residuals are approximately normally distributed (Figures S5 and S6). 

## 4. Discussion

We developed a LUR model for L_den_ computed from continuous five-day noise measurements at 134 outdoor sites in four different areas in Western Cape, South Africa. The LUR model developed contains two road traffic variables (length of big roads within a 200 m buffer, length of medium roads within a 25 m buffer), and three predictor variables related to land use (household density, commercial land use, and industrial land within a 50 m buffer). 

The LUR model demonstrates road traffic to be an essential noise predictor in the study areas, like it was shown by previous studies in Europe and North America [17,21]. However, only 13% of the outdoor noise exposure variability was explained by the LUR model and only 4.7% by the two road traffic variables, which is considerably lower than in these two studies from Europe and North America. There is a good possibility that such a low value is linked to GIS data inaccuracy, including exact geocodes of the measurement sites. Informal settings in South Africa are often constantly changing (new roads and buildings constructions). As a consequence, the GIS data used for this study may not be up-to-date. We may also have missed relevant predictors such as the type of pavement, which is known to affect noise emissions from traffic roads [30,31]. In addition to traffic, the household density was also a significant noise predictor variable. This result was expected because these areas are crowded and, thus, the noise coming from the neighborhood is expected to be more substantial than in Europe and North America. A consequence of the crowding was the absence of low exposure area below 50 dB, which may have further contributed to the low R^2^. Derivation of GIS predictors as a surrogate for neighborhood noise is tricky, since neighborhood noise shows a high temporal variability and is not simply correlated with any geographic predictors. This may be another reason for the low noise variability explained by the LUR model. It would, thus, be interesting to validate our model with source-specific prediction models like the CNOSSOS-EU [32]. However, relevant input data for such a model is not available for this area. Apart from traffic and community noise, both commercial and industrial land use variables were not statistically significant and, thus, less important in our model. The NDVI variable, a surrogate for green spaces, which was the most important variable in the LUR models developed for Montreal, Canada, was not retained in the LUR model for informal settlements [17].

The correlation between predicted and measured noise differed between the four areas. The explained variance was higher in Khayelitsha, and in Marconi-Beam, where noise levels are higher. Khayelitsha is the most crowded area with most of the traffic, followed by Marconi-Beam. This indicates that LUR models are not suitable to model noise exposure in areas with lower environmental noise levels, e.g., from traffic, and presumably more influence from community noise on the noise measurements as in Masiphumulele and Oudtshoorn.

A limitation of this study was the choice of the population, which was selected for a longitudinal cohort study on air pollution and respiratory health outcomes among pupils, and not specifically for noise pollution. Thus, the selected areas are not fully representative for the whole Western Cape including more rural areas. Furthermore, the LUR model was developed based on measurements conducted over a period of six months and not concurrently at all sites. Different results might be obtained for other seasons. For instance, different weather conditions may affect the noise sources, and the noise levels via noise propagation. 

On the other side, this study is the first to give rise to noise measurements over several days in South Africa, more especially in informal settings. In addition, no LUR model for noise exposure had yet been developed with data from the African continent, one previous LUR model having been developed for air pollution in Western Africa [33]. 

## 5. Conclusions

We developed the first LUR model assessing outdoor noise variability in informal settings in Africa. We demonstrated that this method can not only been applied in high-income countries, but also in informal settings. Like in high-income countries, road traffic is an important outdoor noise predictor of the LUR model. Moreover, household density was a significant noise predictor variable. As the population in these locations is dense, the neighborhood noise is considerable. Nevertheless, LUR modeling is more challenging in informal settlements because of constant transformations of these areas, and consequently less accurate GIS data. Furthermore, using household density as a proxy for neighborhood noise may underestimate neighborhood noise and, thus, lead to a weaker LUR model. For this reason, future LUR models may focus on areas primarily exposed to traffic noise. By collecting short-term noise measurements under controlled conditions and avoiding sound from non-traffic sources, one may achieve a higher R^2^ for prediction of road traffic noise. This pioneer study showed interesting results, and encourages further investigation in noise exposure in low- and middle-income countries.

## Figures and Tables

**Table 1:** List of geographic information system (GIS) variables potentially influencing noise levels.

Categories	GIS Variables Description	Unit	Buffer Radius (m)
Roads	Length of large roads	m	25/50/100/200/500
	Length of medium roads	m	25/50/100/200/500
	Length of small roads	m	25/50/100/200/500
	Length of very small roads	m	25/50/100/200/500
	Length of large and medium roads	m	25/50/100/200/500
	Length of large, medium, and small roads	m	25/50/100/200/500
	Length of all roads	m	25/50/100/200/500
	Length of medium and small roads	m	25/50/100/200/500
	Length of medium, small, and very small roads	m	25/50/100/200/500
	Length of small and very small roads	m	25/50/100/200/500
	Inverse distance to nearest road	1/m	
Air	Inverse distance to nearest airport	1/m	
Rail	Inverse distance to nearest railway track in activity	1/m	
	Inverse distance to nearest railway track in activity or not	1/m	
Community	Inverse distance to nearest church	1/m	
	Inverse distance to nearest police station	1/m	
	Inverse distance to nearest hospital	1/m	
Buildings	Household density	# of households/buffer surface in m^2^	25/50/100/200/500/750/1000
Land use	Area of residential land use	m^2^	25/50/100/200/500/750/1000
	Area of commercial land use	m^2^	25/50/100/200/500/750/1000
	Area of industrial land use	m^2^	25/50/100/200/500/750/1000
	Area of buildings land use	m^2^	25/50/100/200/500/750/1000
	Area of nature land use	m^2^	25/50/100/200/500/750/1000
Vegetation	Normalized Difference Vegetation Index (NDVI)	−1 to +1	30/100/150/200/500/750

**Table 2:** Summary statistics (mean, standard deviation (SD), minimum (min), 25th percentile (p25), 50th percentile (p50), 75th percentile (p75), maximum (max)) of the measured noise levels for five different metrics (L_day_, L_evening_, L_night_, L_Aeq24h_, and L_den_), in A-weighted decibels ([dB(A)]).

Variable Name	Mean	SD	Min	p25	p50	p75	Max
L_day_	60.0	4.6	46.1	56.9	60.0	62.9	72.9
L_evening_	60.7	5.1	44.9	57.2	60.2	64.0	77.7
L_night_	52.9	5.5	31.5	49.3	52.6	55.8	72.4
L_Aeq24h_	59.1	4.6	45.1	56.1	58.9	62.0	73.2
L_den_	63.0	4.7	49.1	60.1	62.6	66.5	78.9

**Table 3:** Results of the LUR model developed during a stepwise forward stepwise backward selection, and with only GIS variables in order to explain L_den_ measured at 134 sample sites (adjusted R^2^ = 0.130). The coefficient (coef.) refers to L_den_ increase per unit of the predictor variable.

Variable Name	Buffer Radius (m)	Unit of the Coef. and the (95% CI)	Coef.	(95% CI)	*p*-Value
Households density	50	# of homes/hectare	0.012	(0.004–0.019)	0.003
Length of medium roads	25	m	0.13	(0.05–0.22)	0.003
Length of big roads	200	km	5.4	(−2.15–12.99)	0.159
Area of commercial land use	50	m^2^	0.03	(−0.01–0.07)	0.196
Area of industrial land use	50	100 m^2^	0.12	(−0.04–0.27)	0.145
Intercept		dB	61.30	(60.22–62.39)	<0.001

## Supplementary Materials

The following are available online at www.mdpi.com/1660-4601/14/10/1262/s1, Figure S1: Flowchart of the number of outdoor noise measurements, Figure S2: Box plots of L_day_, L_evening_, L_night_, L_Aeq24h_, and L_den_ measured for each of the four study areas, Figure S3: Box plots of L_den_ measured, and L_den_ predicted (n = 134) for each of the four study areas; Figure S4: Scatterplot between the residuals and their fitted values (n = 134), Figure S5: Distribution of the residuals of the noise predicted values (n = 134), Figure S6: Comparison of the distribution of the residuals of the noise predicted values for each study area separately, Table S1: List of geographic information systems (GIS) variables used to develop the LUR noise model, with their date(s) of establishment and their source, Table S2: List of the variables selected with the univariate analyses to develop the LUR model in order to explain L_den_, with n = 134, Table S3: Summary statistics of the GIS variables use in the LUR model developed to explain L_den_, with n = 134.
