# Supplementary material for: Land Use Regression Modeling of Outdoor Noise Exposure in Informal Settlements in Western Cape, South Africa
Source: Int J Environ Res Public Health. 2017 Oct 20;14(10):1262. doi: 10.3390/ijerph14101262 (PMC5664763; doi:10.3390/ijerph14101262)
Supplement: Supplementary file 1 [file ijerph-14-01262-s001.pdf]

International Journal of
Environmental Research and Public Health

MDPI

Supplementary Materials: Land Use Regression Modeling of Outdoor Noise Exposure Variability in Low Income Areas in Western Cape, South Africa

Chloé Sieber [1,2], Martina S. Ragettli [1,2], Mark Brink [3], Olaniyan Toyib [4], Roslyn Baatjies [4], Apolline Saucy [1,2], Nicole Probst-Hensch [1,2], Mohamed Aqiel Dalvie [4], Martin Röösli [1,2]

List of the Figures and Tables

Figures

Figure S1.	Flow chart of the number of outdoor noise measurements
Figure S2.	Box plots of L_{day}, $L_{evening}$, L_{night}, L_{Aeq24h}, and L_{den} measured for each of the four study areas
Figure S3.	Box plots of L_{den} measured, and L_{den} predicted (n = 134) for each of the four study areas
Figure S4.	Scatter plot between the residuals and their fitted values (n = 134)
Figure S5.	Distribution of the residuals of the noise predicted values (n = 134)
Figure S6.	Comparison of the distribution of the residuals of the noise predicted values for each study area separately

Tables

Table S1	List of geographic information systems (GIS) variables used to develop the LUR noise model, with their date(s) of establishment and their source
Table S2	List of the variables selected with the univariate analyses to develop the LUR model in order to explain L_{den}, with n = 134
Table S3	Summary statistics of the GIS variables use in the LUR model developed to explain L_{den}, with n = 134

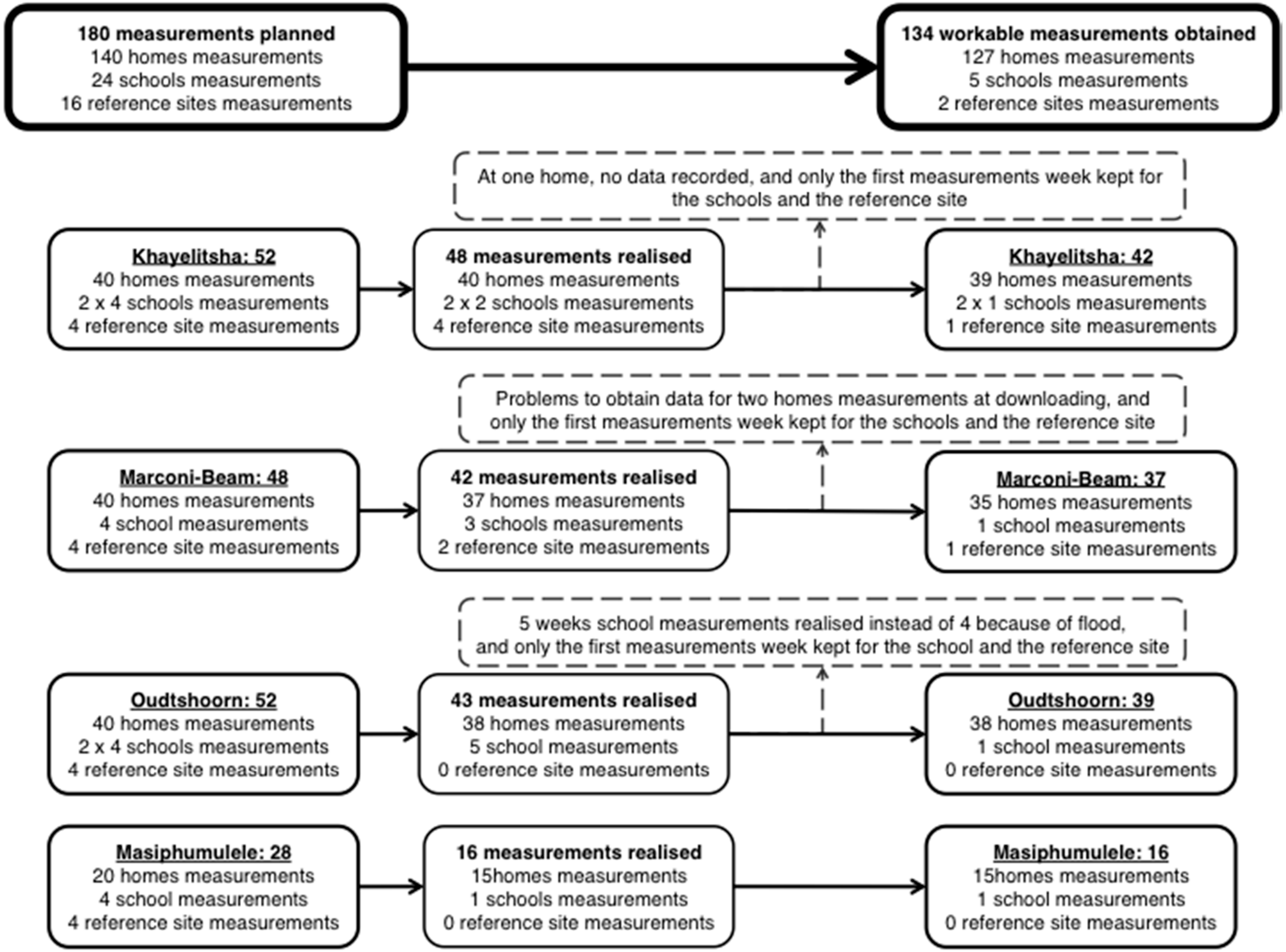

Figure S1. Flow chart showing the number of outdoor noise measurements planned, realized, and used for the analyses. The references sites were difficult to access, therefore so few measurements at these sites were realized.

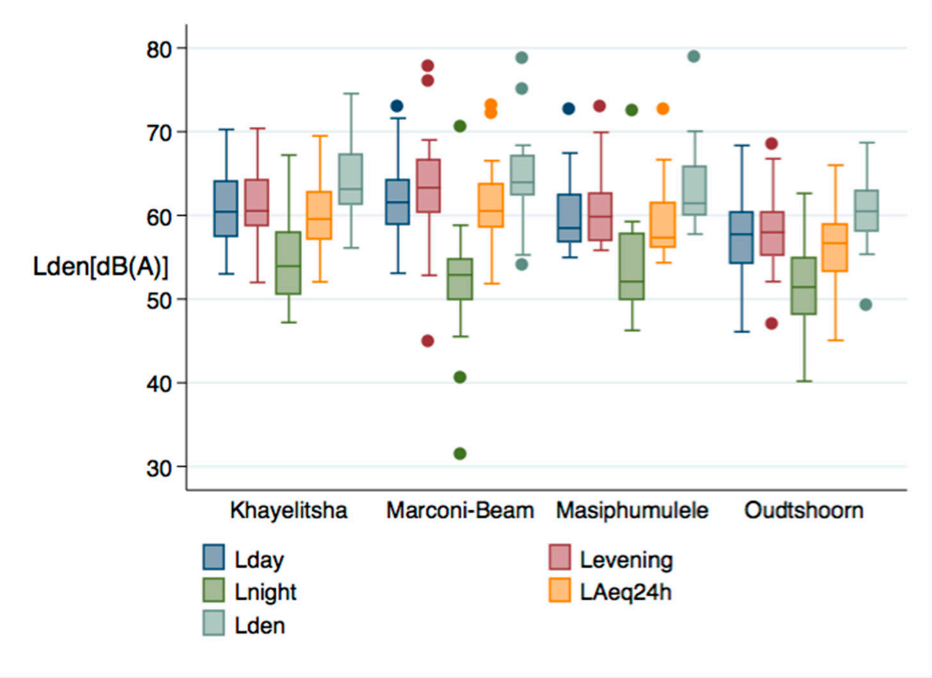

Figure S2. Box plots of L_{day}, $L_{evening}$, L_{night}, L_{Aeq24h}, and L_{den} measured for each of the four study areas (Khayelitsha (n=42), Marconi-Beam (n=37), Masiphumulele (n=16), and Oudtshoorn (n=39)). The boxes represent the interquartile range (IQR), the middle line represents the median, and the whiskers are as long as 1.5 times the IQR. The dots are the values not comprised in the whiskers range.

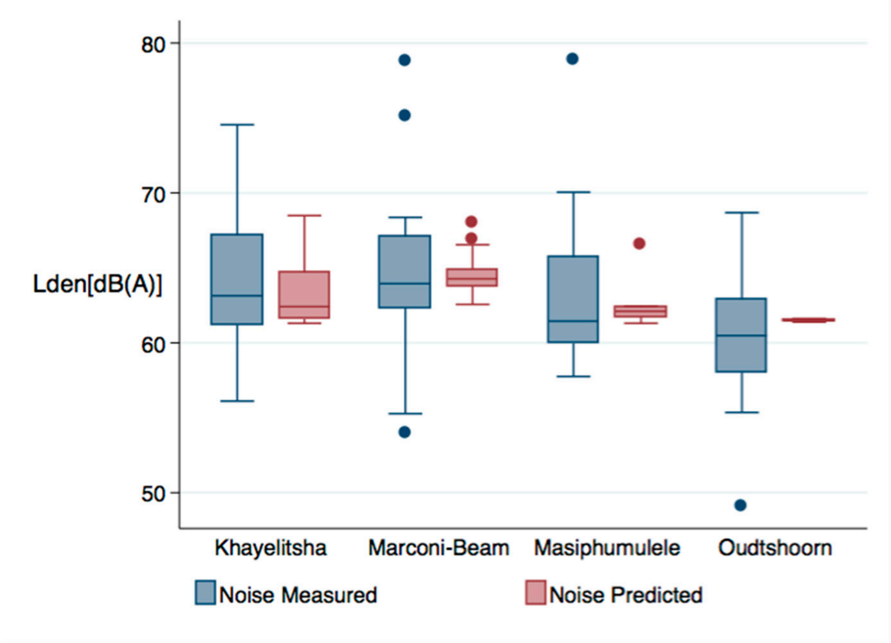

Figure S3. Box plots of L_{den} measured, and L_{den} predicted (n=134) for each of the four study areas (Khayelitsha, Marconi-Beam, Masiphumulele, and Oudtshoorn). The boxes represent the interquartile range (IQR), the middle line represents the median, and the whiskers are as long as 1.5 times the IQR. The dots are the values not comprised in the whiskers range.

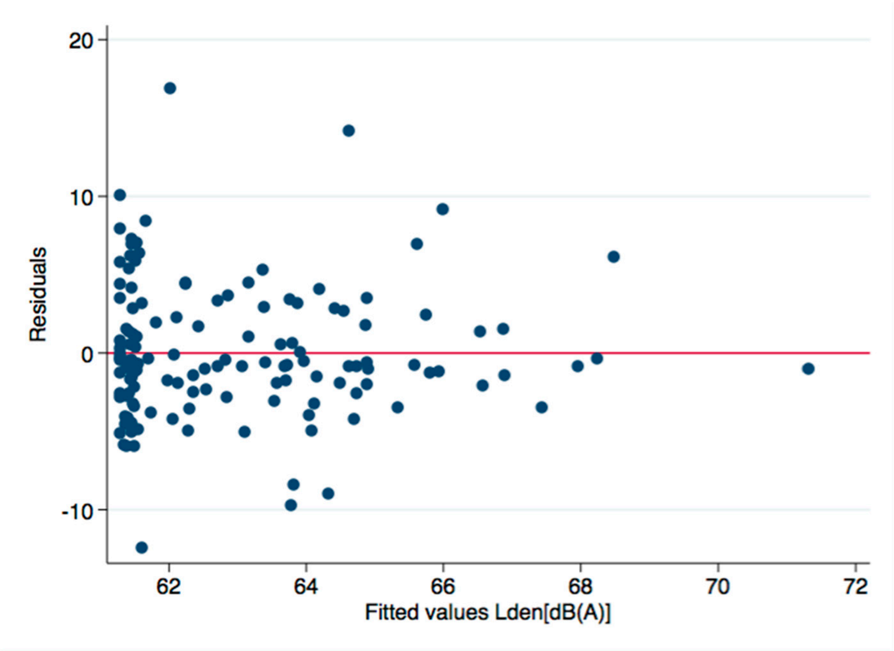

Figure S4. Scatter plot between the residuals and their fitted values, L_{den} in dB (n = 134).

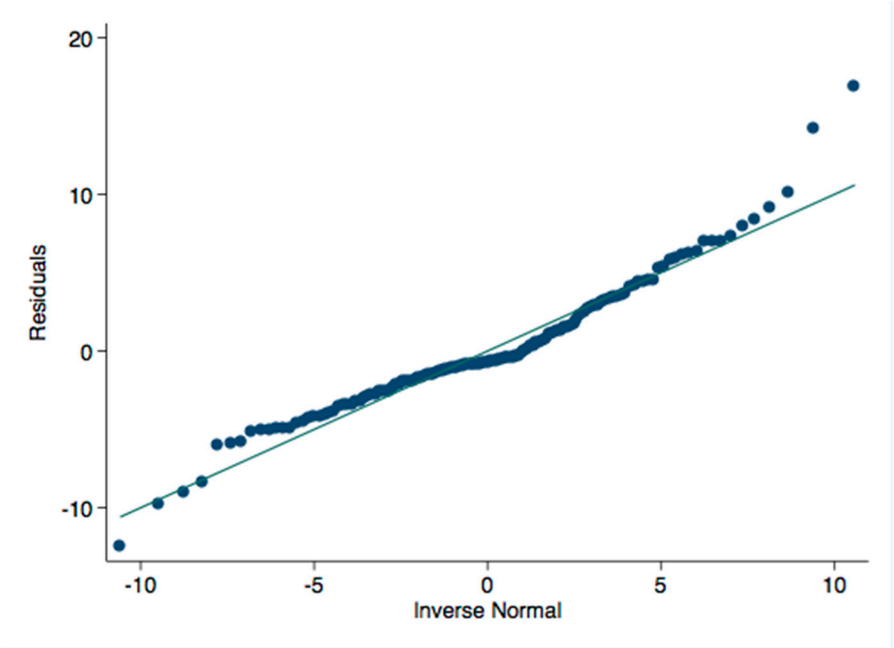

Figure S5. Distribution of the residuals of the noise predicted values (n = 134).

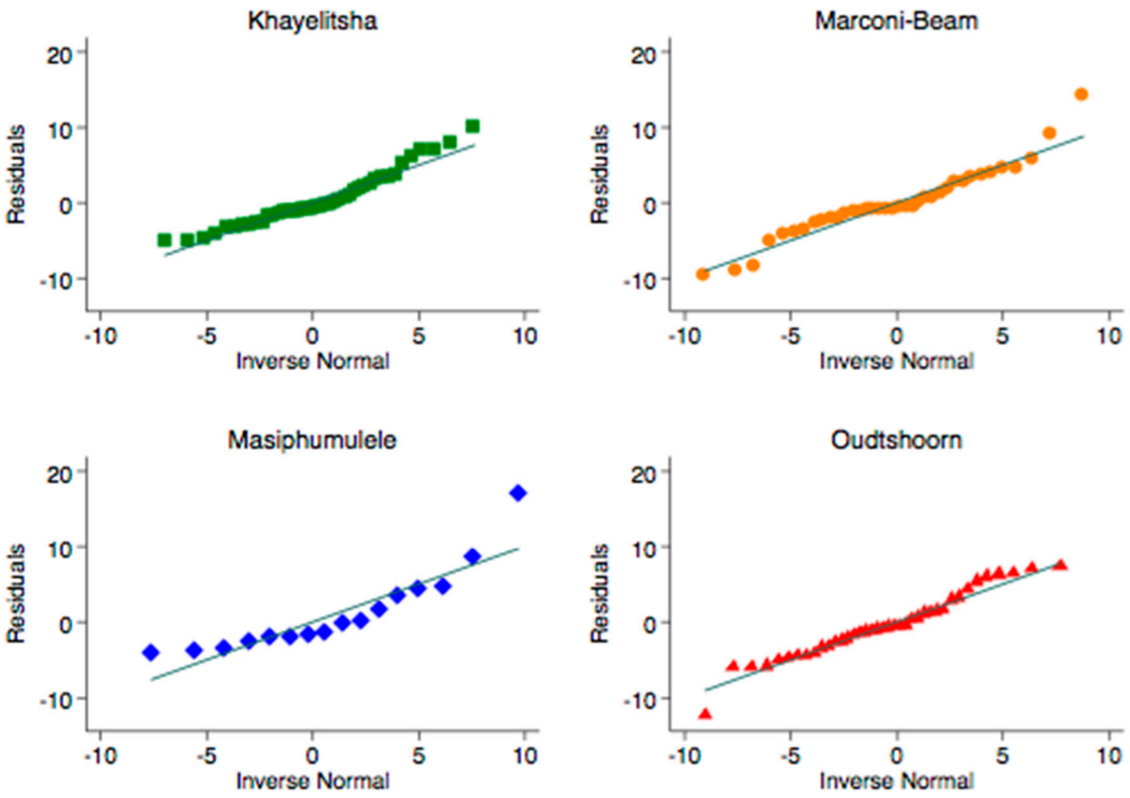

Figure S6. Comparison of the distribution of the residuals of the noise predicted values for each study area separately (Khayelitsha with n = 42, Marconi-Beam with n = 37, Masiphumulele with n = 16, Oudtshoorn with n = 39).

Table S1. List of geographic information systems (GIS) variables used to develop the land use regression (LUR) noise model, with their date(s) of establishment and their source.

Categories	GIS Variables Description	Date	Source
Roads	Roads in Khayelitsha, Marconi-Beam, Masiphumulele	2015	City Maps from the City of Cape Town
	Roads in Oudtshoorn	2011	Municipality of Oudtshoorn
Air	Airport in Khayelitsha, Marconi-Beam, Masiphumulele	2013	City Maps from the City of Cape Town
	Airport/Aerodromes in Oudtshoorn	2009/2016	Municipality of Oudtshoorn
Rail	Railways tracks in Khayelitsha, Marconi-Beam, Masiphumulele	2011	City Maps from the City of Cape Town
	Railways tracks in Oudtshoorn	2009	
Community	Churches in Khayelitsha, Marconi-Beam, Masiphumulele	2014	City Maps from the City of Cape Town
	Churches in Oudtshoorn	2009	Municipality of Oudtshoorn
	Police stations in Khayelitsha, Marconi-Beam, Masiphumulele	2013	City Maps from the City of Cape Town
	Police stations in Oudtshoorn	2009	Municipality of Oudtshoorn
	Hospitals in Khayelitsha, Marconi-Beam, Masiphumulele	2013–2014	City Maps from the City of Cape Town
	Hospitals in Oudtshoorn	2003	Municipality of Oudtshoorn
Buildings	Household density in Khayelitsha, Marconi-Beam, Masiphumulele	2012	City Maps from the City of Cape Town
	Household density in Oudtshoorn	2006-2010	Municipality of Oudtshoorn
Land use	Area of residential land use in Khayelitsha, Marconi-Beam, Masiphumulele	2012	City Maps from the City of Cape Town
	Area of commercial land use in Khayelitsha, Marconi-Beam, Masiphumulele	2012	City Maps from the City of Cape Town
	Area of industrial land use in Khayelitsha, Marconi-Beam, Masiphumulele	2012	City Maps from the City of Cape Town
	Area of buildings land use in Khayelitsha, Marconi-Beam, Masiphumulele	2012; 2013–2014	City Maps from the City of Cape Town

	Area of public parks, natural reserves, water bodies land use in Khayelitsha, Marconi-Beam, Masiphumulele	2012; 2013–2014	City Maps from the City of Cape Town
	Area of residential land use in Oudtshoorn	2006–2010	Municipality of Oudtshoorn
	Area of commercial land use in Oudtshoorn	2009	Municipality of Oudtshoorn
	Area of industrial land use in Oudtshoorn	2010	Municipality of Oudtshoorn
	Area of buildings land use in Oudtshoorn	2009	Municipality of Oudtshoorn
	Area of public parks, natural reserves, water bodies land use in Oudtshoorn	In part from 2009	Municipality of Oudtshoorn
Vegetation	Normalized Difference Vegetation Index (NDVI) in Khayelitsha, Marconi-Beam, Masiphumulele	9 January 2016	U.S. Geological Survey, Earth Explorer, 2016. Available from https://earthexplorer.usgs.gov. Accessed 9 January 2017
	Normalized Difference Vegetation Index (NDVI) in Oudtshoorn	31 March 2016	U.S. Geological Survey, Earth Explorer, 2016. Available from https://earthexplorer.usgs.gov. Accessed 9 January 2017

Table S2. List of the variables selected with the univariate analyses to develop the LUR model in order to explain Lden, with n = 134. The coefficient (coef.) refers to Lden increase per unit of the predictor variable.

Variable Name	Buffer Radius (m)	Coef. and 95% CI unit	Coef.	[95% CI]	R Squared	*p*-Value
Households density	750	# of homes/ km^2 [1]	0.02	[0.00–0.03]	0.050	0.009
Length of big, medium, and small roads	100	m	0.01	[0.00–0.02]	0.048	0.011
Length of medium roads	25	m	0.11	[0.02–0.20]	0.041	0.018
Length of big, and medium roads	500	100m*	0.08	[0.01–0.14]	0.037	0.025
Area of industrial land use	200	10,000m^2*	0.57	[0.05–1.09]	0.035	0.031
Area of commercial land use	50	m^2	0.04	[0.00–0.08]	0.024	0.074
Normalized Difference Vegetation Index (NDVI)	30	−1 to +1	−0.07	[−0.15–0.01]	0.023	0.079
Length of big roads	500	100m*	0.12	[−0.03–0.26]	0.019	0.112
Inverse distance to nearest road		1/m	0.97	[−0.54–2.47]	0.012	0.207
Length of small roads	100	m	0.01	[−0.01–0.03]	0.008	0.291
Area of nature land use	25	100m^2*	−0.14	[−0.42–0.14]	0.008	0.319
Length of all roads	25	m	0.01	[−0.02–0.03]	0.001	0.671

[1] The unit of the coefficient value, and the 95% CI were adapted in order to produce readable values.

Table S3. Summary statistics of the GIS variables use in the LUR model developed to explain Lden, with n = 134.

**Variable Name**	**Buffer Radius (m)**	**Unit of the Variable**	**Mean**	**SD**	**Min**	**p25**	**p50**	**p75**	**Max**
Households density	50	# of homes/ hectare	96.6	107.2	0	10.3	33.5	195.1	375.8
Length of medium roads	25	m	2.2	9.0	0.0	0.0	0.0	0.0	49.3
Length of big roads	200	km	0,021	0.115	0.0	0.0	0.0	0.0	0.822
Area of commercial land use	50	m^2	2.6	20.3	0.0	0.0	0.0	0.0	223.4
Area of industrial land use	50	$100m^2$	1.12	4.99	0.0	0.0	0.0	0.0	43.58

© 2017 by the authors. Submitted for possible open access publication under the terms and conditions of the Creative Commons Attribution (CC BY) license (http://creativecommons.org/licenses/by/4.0/).
